# Mapping accessibility to oral health care in coastal India – A geospatial approach using a geographic information system (GIS)

**DOI:** 10.12688/f1000research.75708.2

**Published:** 2022-08-08

**Authors:** Prajna Pramod Nayak, Soham Mitra, Jagadeesha B. Pai, Ramprasad Vasthare Prabhakar, Nandita Kshetrimayum

**Affiliations:** 1 Department of Public Health Dentistry, Manipal College of Dental Sciences, Manipal Academy of Higher Education, Manipal, Karnataka, 576104, India; 2 Manipal College of Dental Sciences, Manipal Academy of Higher Education, Manipal, Karnataka, 576104, India; 3 Department of Civil Engineering, Manipal Institute of Technology, Manipal Academy of Higher Education, Manipal, Karnataka, 576104, India; 4 Dept. of Public Health Dentistry, Dental College, Regional Institute of Medical Sciences, Imphal, Manipur, 795004, India

**Keywords:** Geographic Information Systems, spatial analysis, geographic accessibility, oral health services.

## Abstract

**Background: **It is imperative to have a thorough assessment of the existing distribution of oral healthcare facilities and understand potential accessibility when planning for expansion of oral health services. In the present study, an attempt to measure geographic accessibility to oral healthcare, by locating the availability of dental practitioners in the coastal districts of Karnataka state, India using a geographical information system (GIS), has been made.

**Methods: **For the study, data on public and private oral health centres were collected for the three coastal districts of Karnataka state, India. Population and income data were collected, along with geographic attributes (latitudes and longitudes) of the practitioners' addresses. Descriptive statistical analyses and dentist-to-population ratios (D:P) were calculated. Correlation between the number of clinics with population and D:P with per capita income were analyzed using Pearson's correlation coefficient. Chi-square test applied to analyze any association between D:P and urbanization.

**Results:** Among 340 clinics, 8.5% are public and 91.5% are private clinics catering to a population of 4,704,179. Average D:P for the three coastal districts is 1:13,836. There is an uneven urban-rural distribution of dentists with lower D:P in rural areas. Rural population in four taluks have only one dentist for over a lakh population. Six taluks have only one dentist for every 50000 – 100000 population in rural areas. Six rural areas had only public centers to cater to their oral health.

**Conclusions:** From the study, it is concluded that oral health services were concentrated in areas with higher annual income per-capita, increased urbanization and population density.

## Introduction

Health For All has become a corner-stone of public health, ever since the International Conference on Primary Healthcare and declaration of Alma Ata in 1978. Health for all is not just being free from diseases; it is a promise for the provision of basic services to every single person in the world. Right to healthcare is a basic human necessity and includes dental healthcare within its umbrella of holistic cares. ^1^

One of the principles of primary health care is to provide social equity universally. The word equity refers to just and fair distribution of health care services all around the globe. ^1^ There is a special emphasis on providing access, which is people’s ability to avail healthcare related services as and when they are needed. ^2^ Access to healthcare has an important role to play in the overall health system, in reducing the burden of disease. ^2^ Access to health care has two important geographical perspectives:

1. Accessibility (potential of approach) – how conveniently a particular service can be approached and the means of accessing the facility; and
2. Utilization (actual frequency of approach) – actual use of services at hand.

It is very well acknowledged that, the healthcare based resources need to be planned in a specific way that, they are utilized to the maximum. ^3^ They need to be located conveniently for the majority of the population to be able to access them. It is also important to accommodate for different growth rates of different geographic areas and population clusters. Some areas are dynamic and mutate, evolve and grow with time. This uneven growth rate of population clusters is straining an already stressed utility provision system. The prime objective for healthcare providers is to have an adequate road map during the establishment of new healthcare centres so that it caters to maximum number of people. ^3^

Given the above criteria, geographic information system (GIS) based accessibility interpretation is the most scientific and succinct method that can be used to calculate the extent to which geographical access is obtained. ^4^^,^^5^ In short, GIS is the ‘digitization of cartogram’. It is a modern information system with capabilities of accepting, recording, analysing, managing and presenting the spatial referenced data (that is, the data is linked to a geographic location). ^6^ It uses data that is attached to a unique location (geo-referencing) to create a multi-tier map showing individual attributes that can be superimposed. GIS allows and aids in cartographical representation and comparison of data that can be used in formulating better and focused healthcare plans. Pictorial representation of statistical data is much more lucid and easier to interpret in comparison to other formats of data representation. ^7^ Density maps/Heat maps aid in finding density of health centres, cases, vectors, risk factors, etc. Kernel density calculates the density of features in a neighbourhood and around those features, per unit area, in a raster format. It can be used with both the point and line data. ^7^

In terms of geographical extent, India enjoys the position of being the seventh largest country worldwide, with a population of 1.3 billion. In India, dental healthcare is provided by a combination of private sector and public institutions. As one of the major signatories of the United Nations (UN) charter for health, India is committed to provide basic dental health provision, starting at the level of Community Health Centres. Since the inception of India’s first dental college in Kolkata in the year 1920, it has been an uphill task to train sufficient number of dentists to meet the demands of an ever-growing population. Annual government funding for health services is meagre 1.6% of our total GDP, with no separate allocation for oral health. ^8^ With 22% of the population living below poverty line, providing basic dental health services is a mammoth task. ^9^

Coastal Karnataka includes the districts of Udupi, Dakshina Kannada and Uttara Kannada in the South Western part of India. This area has its own ethnic population and customs and boasts of a population of roughly 4.7 million, spread over an area of 18730 square kilometer. The settlements vary greatly from commercial and urban clusters of Mangalore to inaccessible villages of Dandeli. This uneven distribution of population, and living standards warrants individual surveys for planning. (GIS)-based model is been applied in other countries to estimate the number of new oral health facilities needed based on the geographical proximity or distance to nearest health care facility. ^3^^,^^6^^,^^10^

This thorough assessment of the existing distribution of oral healthcare facilities to understand potential accessibility when planning for expansion of oral health services. We hypothesized that dentists are concentrated to areas with high population density, easy geographic access and a higher wealth distribution.

Hence, this study aimed to map and calculate objectively, spatial accessibility to the oral health care facilities in coastal districts of Karnataka state, India: Udupi, Uttara Kannada and Dakshina Kannada, and its association with population density and socio-economic conditions. Objectives were:

1. to analyze the geographic distribution of private and public dental healthcare providers with respect to population, per-capita income and urbanization, in GIS environment;
2. to identify the poorly served areas in the three coastal districts of Karnataka and
3. to investigate any association between oral health care services and socio-economic conditions.

## Methods

**Figure 1.**
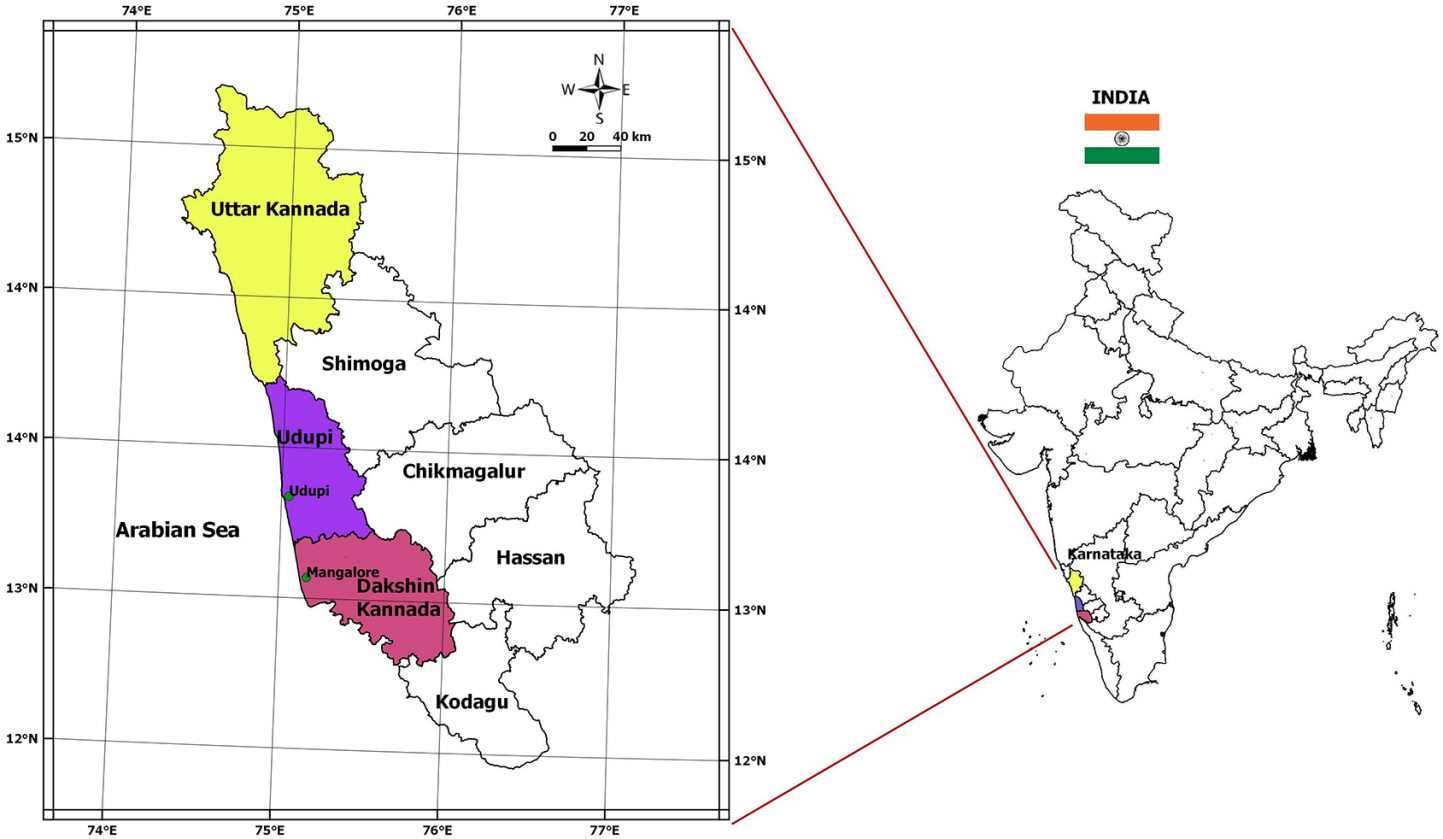
Location map of study area.

The three coastal districts of Karnataka stretch to a length of 435 km and a width of 225 km. These Districts are further divided into administrative sub-units known as taluks. There are a total of 1807 villages in 20 Taluks of these 3 Districts. We chose administrative districts as our geographical regions, since these match the population census files in the same format, as required for geo-mapping and analysis of geographical data. This cross-sectional study was conducted over a duration of twelve weeks between September and November 2020. Figure 1 gives the location map of the study area, with reference to India.

### Collection of data on public and private oral health services

Indian oral health care delivery system comprises predominantly of a private sector and a smaller public sector. Data on the public and private oral health centres were collected.

Public health care centers: Oral health delivery in public sector is integrated into the existing network of public hospitals in India and is organized in a hierarchy based on administrative units and population size. Oral healthcare is provided by Community Health Centres (CHC’s), district hospitals and government teaching institutions spread across the country. The address of each of these centers with dental clinics were obtained from the official portal of Karnataka State Health Ministry. ^11^

Private dental clinics: Private delivery system has been the predominant format of care in India. There is no official database maintained for the private dental clinics in our country. Therefore, the addresses were obtained from the largest dental non-governmental organization in India (Indian Dental Association branches of the three districts). We also hand-searched for any other private clinics through Google Search and advertisements.

Teaching Dental Hospitals: Addresses of all the private dental schools with attached tertiary hospitals available in these districts were also included.

To test for accuracy of the geocoded dental practices, 2% of all practices with geocoding were chosen randomly and tested with Google Maps and open street maps and further web searched to attest to the authenticity of the data.

### Population statistics & socioeconomic data

Population based statistics were obtained from the most recent National Census of India (2011). ^12^ Urban-rural divide of the population was obtained from Primary Census Abstract Data Highlights of Karnataka. ^13^

Per-capita income at the district level was extracted from India Human Development Survey II (2011-12). ^14^

### Geo-coding

The geographic extent of the study area is from 15.5252°N to 12.8437°N latitude and 74.0852°E to 75.2479°E longitude covering an area of 18,931 square km. Geo-coding of the all the public and private oral health care services were completed by plotting on the free access geo-coding website from Google (OpenStreetMap). Geographic attributes (latitudes and longitudes) were then designated to the practitioners’ addresses with 90% being at the level of the building. Exclusion of duplicate addresses were done. After recording and cleaning, database files were transferred to the Quantum Geographic Information Systems (QGIS version 3.14, QGIS Development Team, GNU General Public License, Essen, Germany), with World Geodetic System 1984 (WGS 84) standard of coordinate referencing for geo-mapping.

### Statistical analyses

The extracted data sets were then transferred to Microsoft Excel 2010 (v 2019, Microsoft Redmond Campus, Redmond, Washington, United States). Data was subjected to statistical analysis using Statistical package for social sciences (SPSS v 26.0, IBM). Descriptive statistical analyses (including number of dental practices within each district) and dentist-to-population ratios (D:P) were calculated. Correlation between the number of dental clinics with population and D:P with per capita income were analyzed using Pearson’s Correlation coefficient. Chi-square test was applied to analyze any association between D:P and urbanization.

## Results

### Geographic distribution of dental clinics

We located 340 clinics functioning currently in the 20 taluks of the 3 districts, 29 (8.5%) of which are public and 311 (91.5%) are private clinics and cater to a population of 4,704,179. ^29^ Overall, there are 255 (75%) urban and 85 (25%) rural clinics in the three districts as shown in Table 1.

**Table 1.** District-wise distribution of dental workforce by practice type and location.

District	Total No. of dental clinics	Total population	Private clinics	Public clinics	Dental schools	Urban clinics	Rural clinics	Urban population	Rural population
Uttara Kannada	67	1437169	59	8	0	42	25	418981	1018188
Udupi	109	1177361	101	8	1	74	35	334061	843300
Dakshina Kannada	164	2089649	151	13	5	139	25	996086	1093563
**Total**	**340**	**4704179**	**311**	**29**	**6**	**255**	**85**	**1749128**	**2955051**

Among the 11 taluks in Uttara Kannada, almost all (n=9) have less than 10 clinics and 10-20 clinics in 3 taluks. In contrast, one taluk each of Udupi and Dakshina Kannada districts have 40-50 clinics as shown in Table 2.

**Table 2.** Taluk-wise distribution of dental clinics stratified according to the 3 districts.

District	Number of clinics in a taluk					Total
	≤ 10	11-20	21-30	31-40	≥ 40	
Uttara Kannada	9	3	0	0	0	12
Udupi	0	1	0	1	1	3
Dakshina Kannada	2	0	2	0	1	5

Average D:P for the three coastal districts is 1:13,836. Udupi district has lowest D:P of 1:10801, followed by Dakshina Kannada with ratio of 1: 12742 and Uttara Kannada with highest D:P of 1: 21450. Taluk-wise ratios showed lowest D:P in Mangalore taluk (1:9656) while Mundgod has the highest D:P (1:53087). Mangalore has the highest number of dental clinics (N=103), of which 96 (93.2%) were private clinics, which is also the taluk with highest per-capita income. Likewise, Mundgod taluk that has highest D:P (1:53087) has lowest per-capita income as shown in Table 3.

**Table 3.** Taluk-wise D:P and per-capita income.

District	Taluk	No. of clinics	Total population	D:P ratio	Per capita income
Uttara Kannada	Karwar	12	155213	12934	34259
	Supa	1	52012	52012	27812
	Haliyal	4	119357	29839	33966
	Yellapur	2	78662	39331	26813
	Mundgod	2	106174	53087	44326
	Sirsi	11	186908	16992	29742
	Ankola	4	107332	26833	30086
	Kumta	13	154280	11868	44899
	Siddapur	3	97322	32441	30989
	Honavar	6	166264	27711	27216
	Bhatkal	6	161576	26929	44251
	Dandeli	3	52069	17356	51765
	**Total**	**67**	**1437169**	**21450**	**35767**
Udupi	Kundapura	39	398471	10217	73676
	Udupi	55	562799	10233	60920
	Karkal	15	216091	14406	94716
	**Total**	**109**	**1177361**	**10801**	**62120**
Dakshina Kannada	Mangalore	103	994602	11051	54572
	Bantval	26	395380	15207	50267
	Beltangadi	9	266589	44432	53245
	Puttur	21	287851	13707	81882
	Sulya	5	145227	29045	34259
	**Total**	**264**	**2089649**	**13533**	**66936**
**Total**		**340**	**4704179**	**13836**	**54941**

D:P – Dentist-to-population ratio.

**Figure 2.**
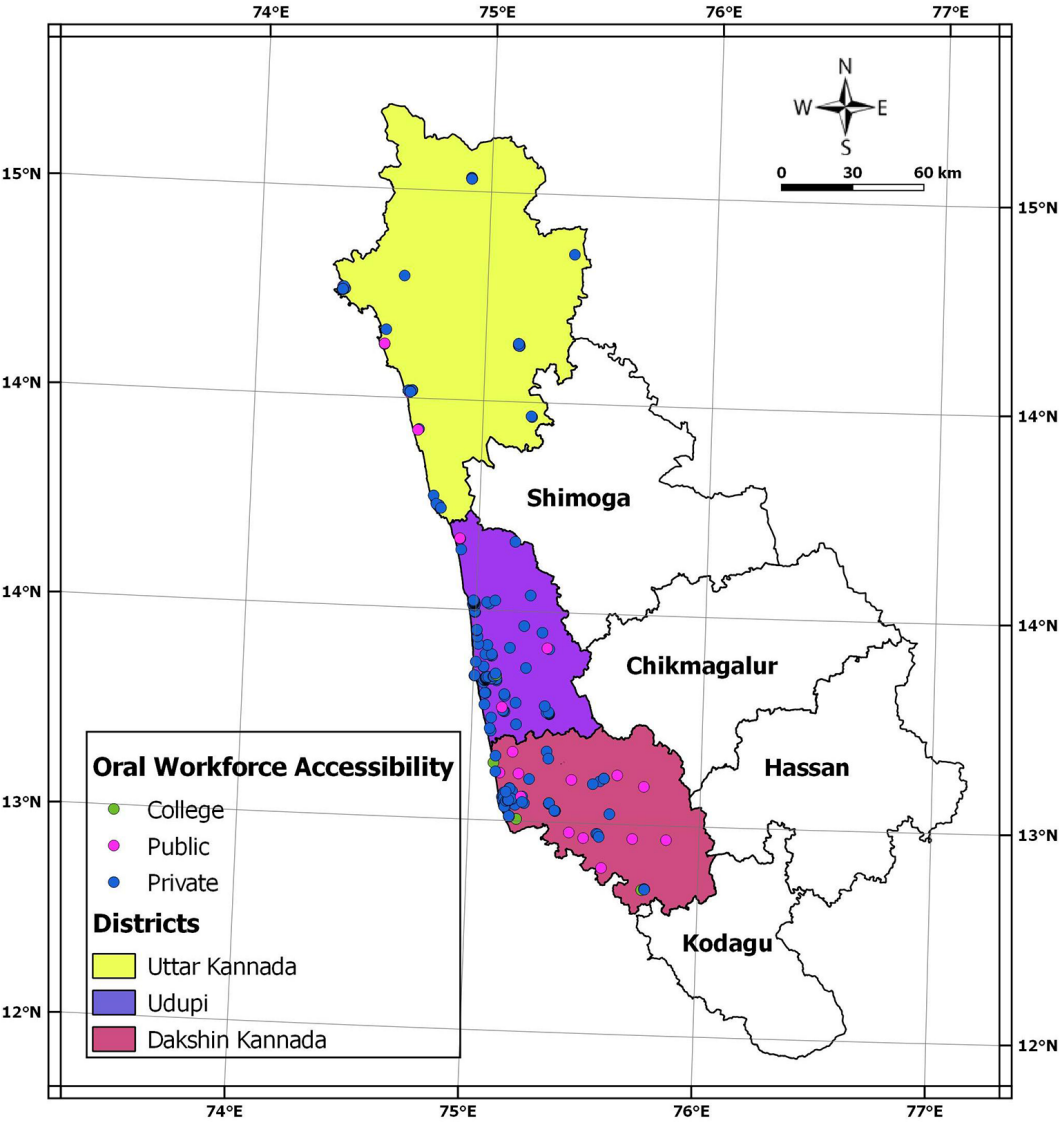
Geo-mapping of the private and public oral health care facilities and Dental school distribution in the three coastal districts of Karnataka.

Figure 2 gives the geo-map for the three coastal districts. Bourgeoning of private dental clinics and dental schools in a few areas is distinctly evident. Out of 340 clinics in the three districts, 103 were situated in Mangalore taluk. Further, Mangalore taluk alone houses five dental schools of the total seven dental schools in three districts. Uttara Kannada district does not have any dental schools.

### Identification of poorly served areas

**Figure 3.**
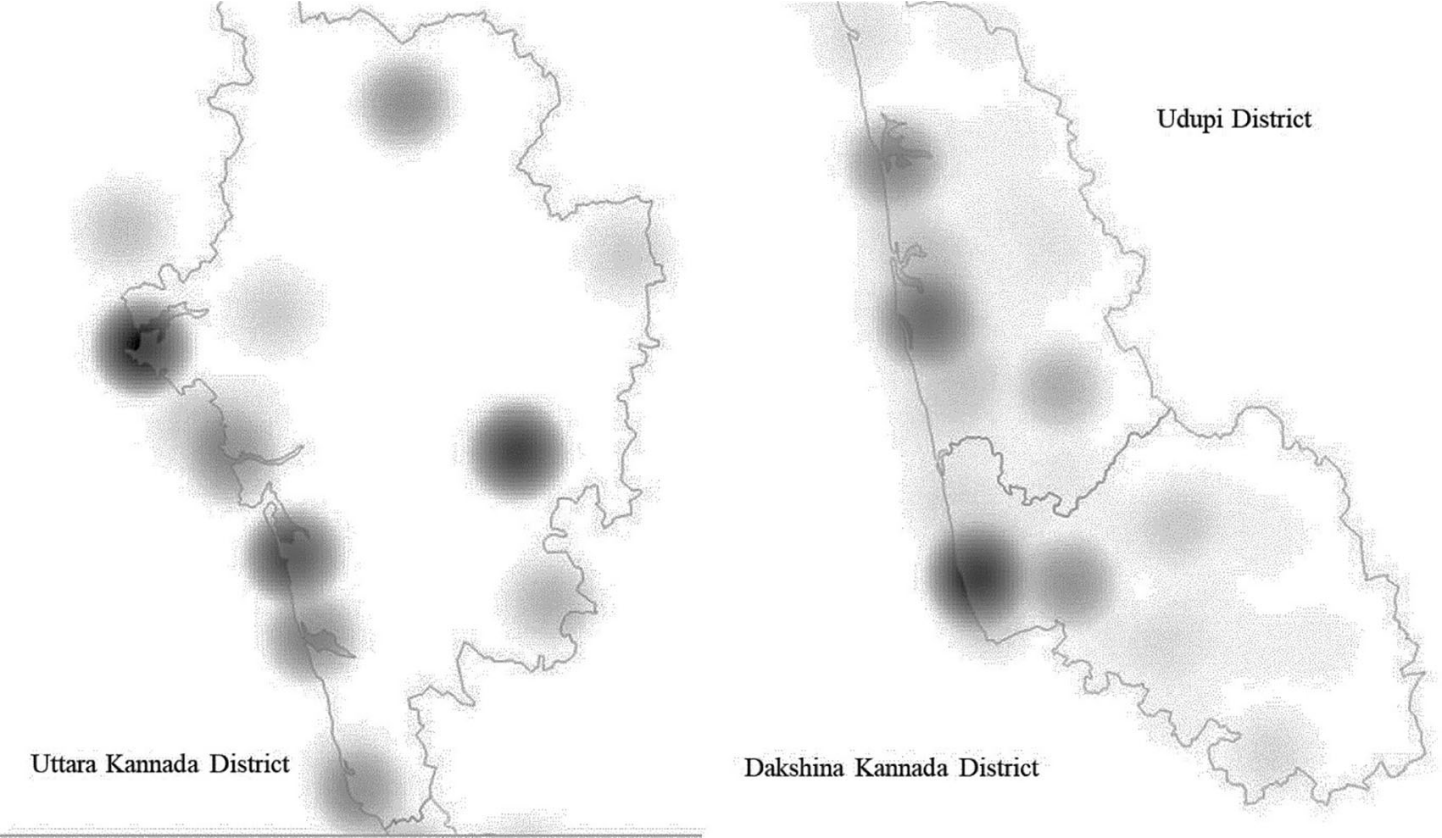
Heat map/Density Map showing the concentration of Oral health care facilities of 3 districts.

There is an uneven urban-rural distribution of dentists with lower D:P in rural areas. Rural population in four taluks have only one dentist for over a lakh population. Six taluks have only one dentist for every 50000 – 100000 population in rural areas. Six rural areas had only public centers to cater to their oral health. Highest concentration of dental schools was seen in Dakshina Kannada district, with four schools in Mangalore taluk. Uttara Kannada district has no dental schools, in spite of being the biggest district of the three ( Table 3). Identification of poorly served areas in the three coastal districts of Karnataka was facilitated with the aid of heat maps/density maps as shown in Figure 3. Kernel Density Estimation algorithm is used for creating heat map. This method is basically a Probability Density Function (PDF) which estimates or predicts an unknown value at a location from known value by means of interpolation.

### Association of dental clinic distribution with population, urbanization and socio-economic conditions

Significant positive correlation is seen between taluk-wise population and number of dental clinics (Pearson’s correlation coefficient = 0.984) and also with D:P and per-capita income (Pearson’s correlation coefficient = -0.548). Chi-square test applied to determine association between D:P and urbanization is found to be significant (p < 0.000).

## Discussion

Universal healthcare facilities are one of the pillars of the healthcare planning process. Access to healthcare is different from the geographic accessibility, in that, the former encompasses both:

1. spatial components (availability and accessibility) and
2. aspatial components (acceptability and affordability).

Hence, in our study we tried to measure geographic accessibility by locating the availability of dental practitioners in the coastal districts of Karnataka using GIS. A geographic information system (GIS) is a computerized system, that is created to analyse and display geographically referenced information as a layered map 6. According to Padminee K *et al.*, Karnataka has the largest number of Dental practitioners for any state in India (34,768). ^15^ For our work, geo-coordinates of the public and private oral health centres were collected and this data was used for GIS analysis using the open-source software, QGIS 3.14.

In the present study the average D:P was 1:12836. This is in accordance with the national D:P of 1:10,271. ^16^ World Health Organization (WHO) recommendations state the ideal dentist to population ratio should be 1:7500. ^17^ The above data is in sync with that of other developing countries. In India there are more than 300 dental colleges with 24,000 dental graduates adding to the pool every year. This ratio is similar to the studies conducted by Periera I *et al.* and Hosny G *et al.* in Srilanka and Egypt, respectively, ^17^^,^^18^ but more than that of the study conducted by Omogunloye OG *et al.* ^19^ but much lesser than the ratio reported in studies conducted in Australia and the United States (US). ^20^^,^^21^

There is a severe misallocation of dentists in terms of urban and rural distribution. A high proportion of dental professionals are concentrated in the urban agglomerations. Only a small Indian population of 15 – 20% have access to dental health services through national schemes. The average per capita public health funding for a year in India is a meagre 2.6$. ^15^

According to the current study, a meagre 25% of the dental practitioners were practicing in the rural areas, serving 37.2% of coastal population, making D:P in the rural areas to 1: 39,401. This is very similar to the national estimates of rural D:P of 1:30,000. ^16^ This contrasts with an urban D:P of 1:6859. These values are very similar to the national urban D:P of 1:4,000. ^16^ This distribution pattern is common to many countries. Brazil and Taiwan (as developing nations) report an average D:P of 1:735 and 1:1603, respectively, but show uneven distribution between urban and rural areas. ^22^^,^^23^ The solution for this uneven distribution lies in bringing all dentists under the umbrella of primary health-care system. In India, providing dental care starts at the level of community health centers. Employment of dental manpower at the primary health center level can help reduce this burden. The dental practitioner to population ratio has markedly improved from 1:301,000 in the 1960s to 1:9992 in the present times and yet, the state-wise distribution of dentists is disproportionate. ^24^

Public health centers are providing affordable oral care services, but the services are very limited. This in turn, compels the people to consult private health care facilities, resulting in excessive expenditures. This situation is the same as in other developing countries like Nepal, Taiwan and Brazil. ^22^^–^^24^ In Nepal the current ratio is 1:16000 according to a population report by Central Bureau of Statistics. Only 8% of dentists work in public sector, and these values are even lower in comparison to countries like Denmark and South Africa. Though the ratio has improved to 1:24000 in 2010 from 1:120000 in 2000, it is much lesser when compared to countries like Singapore and United Kingdom. ^25^ These countries provide holistic dental care via the National Health Service and have a strong network of public health centres for all its citizens. ^26^

Since the 1990s, there has been a boom in the number of dental schools, most of which are private. At present, 86% of all Indian dental schools are private colleges. We observed dissimilarity in the distribution of dental schools across the region. Four dental schools, all private, are located in a single taluk, with one district devoid of any dental schools. Privatization increases the divide between rich and poor, boosting the facilities richest while driving the poor to further penury. ^26^ This should not be acceptable to a civilized society. This wave of privatization has side-lined access to universal oral health services and has alienated the underprivileged. ^27^

The results of our study confirm the hypothesis that dentists are concentrated to areas with high population density, easy geographic access and a higher wealth distribution. This disparity in distribution of oral healthcare amongst the districts of Coastal Karnataka is similar to that of other countries, with dental services being more easily found in the large cities and along the coastal areas. ^6^^,^^23^

Our study has some limitations. Comprehensive data on the dental clinics were not available. Many countries around the world have an annually updated database of dentists in both private and public and sectors. The Indian database is substantially inadequate in this regard. For this study we collected all the data on private dental clinics via the registered dentists under Indian Dental Association. We also hand searched for any private clinics via Google search engine. The latest population data available to us was that of the National Census 2011, whereas, the dental clinics data is updated to 2019 and hence, there are possibilities of overestimation of clinics/D:P ratio. Also, the study cannot envisage the differences that may occur in the dental services utilization among urban and rural populations.

## Conclusion

From our study, we came to the conclusion that oral health services were concentrated in areas with higher annual income per-capita, increased urbanization and population density. The same were unevenly spread across coastal districts. Rural population in four taluks have only one dentist for over a lakh population. Most of the rural areas have only public centres to cater to their oral health. Private clinics are unevenly distributed. Also, we could identify the poorly served areas in the three districts. Shockingly, the share of funds allocated for public healthcare provision is only 1% of the total GDP. Moreover, India lacks a separate and specific allocation for dental health. In the last financial India spent only 6% of its total GDP towards healthcare. ^28^

We anticipate that above problems can be solved with appropriate governmental support by the establishment of public health centers in inaccessible areas. Meticulous planning of survey data comprising of geographical distribution parameters and economic status of the surrounding population can give an accurate representation of the ease of accessibility of treatment.

## Data availability

### Underlying data

Open Science Framework: https://doi.org/10.17605/OSF.IO/A8SNJ. ^29^

License: Data are available under the terms of the Creative Commons Zero “No rights reserved” data waiver (CC0 1.0 Public domain dedication).
