# Supplementary material for: Mapping accessibility to oral health care in coastal India – A geospatial approach using a geographic information system (GIS)
Source: F1000Res. 2022 Aug 8;11:366. Originally published 2022 Mar 30. [Version 2] doi: 10.12688/f1000research.75708.2 (PMC9363979; doi:10.12688/f1000research.75708.2)
Supplement: Peer review history

# Peer review: Reviewer response for version 2

**DOI:** 10.5256/f1000research.137142.r146901

**Published:** 2022-08-09

**Authors:** Arun Pallathadka

**Affiliations:** 1 Department of Geography, Portland State University, Portland, OR, USA

The authors have addressed my previous comments well. I approve the revised version of the work for indexing.

Is the work clearly and accurately presented and does it cite the current literature?

Partly

If applicable, is the statistical analysis and its interpretation appropriate?

Yes

Are all the source data underlying the results available to ensure full reproducibility?

Yes

Is the study design appropriate and is the work technically sound?

Yes

Are the conclusions drawn adequately supported by the results?

Yes

Are sufficient details of methods and analysis provided to allow replication by others?

Yes

Reviewer Expertise:

I specialize in GIS, Spatial Analysis, Spatial Statistics, Urban Geography, and Sustainability.

I confirm that I have read this submission and believe that I have an appropriate level of expertise to confirm that it is of an acceptable scientific standard.

# Peer review: Reviewer response for version 1

**DOI:** 10.5256/f1000research.79617.r145194

**Published:** 2022-07-28

**Authors:** Arun Pallathadka

**Affiliations:** 1 Department of Geography, Portland State University, Portland, OR, USA

This work uses GIS methods to analyze accessibility to dental services in 20 taluks of 3 districts in Karnataka, India. The authors have geocoded dental practitioner locations using open resources such as Google Maps and OpenStreetMap, and have further relied on density mapping and Pearson's correlation to report their findings. Overall, this work has merit, especially in highlighting the urban-rural divide in dental service accessibility in India. While the authors have shown promising potential in the area of geographic information system (GIS) applications, I note that there are sections in this manuscript that could be improved. Following are my comments for improvement:

1. In the introduction section, the authors introduce healthcare accessibility well but do not discuss the state of knowledge on dental care accessibility. Discuss previous works summarizing what similar studies (global, regional, local) have found. I have added references that may be helpful to the authors. 
2. I would recommend not using 1 sentence paragraphs in the manuscript. For example, "socioeconomic data" has only one sentence. You can either add a few more sentences to such subsections or combine them into a single section under appropriate headings. 
3. I would strongly encourage the authors to include a site map showing the location of the three districts with all the major cities clearly shown, and then also show a Karnataka map (with just district boundaries) within the Indian map (state boundaries only). Include the Arabian sea. 
4. Revise **Fig. 1** with the following changes: 
  - Fix the legend heading, i.e., " *Selected_points*" is unnecessary. Figures should be clean without underscores.
  - Under three districts on the legend, it is not clear what the blue polygon represents as it stands without a label.  
  - I would also include a very light base map of neighboring districts and the Arabian Sea for reference. 
5. In the methods, briefly describe what technique (E.g., Kernel) / tool (E.g., Point Density tool) was used for density/heat mapping analysis. 
6. It is not clear what software was used to run Pearson and Chi-square tests. Please include the appropriate citations. 
7. I am not sure this paragraph fits into the scope of discussion. It would better fit in the methods section or perhaps even the introduction section where the study area is described.
*" Hence, in our study we tried to measure geographic accessibility by locating the availability of dental practitioners in the coastal districts of Karnataka using GIS. A geographic information system (GIS) is a computerized system, that is created to analyse and display geographically referenced information as a layered map 6. According to Padminee K et al., Karnataka has the largest number of Dental practitioners for any state in India (34,768). ^14^ For our work, geo-coordinates of the public and private oral health centres were collected and this data was used for GIS analysis using the open-source software, QGIS 3.14."*
* *
8. In your limitations, also acknowledge the differences that may exist in dental service preferences among urban and rural populations. This applies to both dentist and patient behaviors. 
9. In your conclusion section, refer to the sentence I have provided below. The statement is too strong/broad. Perhaps, rephrase to state how these problems can be better addressed with appropriate government support, along with support from private and non-governmental stakeholders. 
*"All the above mentioned problems can be solved with appropriate government support in key areas."*

Is the work clearly and accurately presented and does it cite the current literature?

Partly

If applicable, is the statistical analysis and its interpretation appropriate?

Yes

Are all the source data underlying the results available to ensure full reproducibility?

Yes

Is the study design appropriate and is the work technically sound?

Yes

Are the conclusions drawn adequately supported by the results?

Yes

Are sufficient details of methods and analysis provided to allow replication by others?

Yes

Reviewer Expertise:

I specialize in GIS, Spatial Analysis, Spatial Statistics, Urban Geography, and Sustainability.

I confirm that I have read this submission and believe that I have an appropriate level of expertise to confirm that it is of an acceptable scientific standard, however I have significant reservations, as outlined above.

## References

[1] : Analyzing location-based accessibility to dental services: an Ohio case study. *J Public Health Dent* .2007;67(2) : 10.1111/j.1752-7325.2007.00027.x 113-8 10.1111/j.1752-7325.2007.00027.x 17557683

[2] : GIS mapping of healthcare practices: do older adults have equitable access to dental and medical care in the UK?. *Br Dent J* .2021; 10.1038/s41415-021-3406-0 10.1038/s41415-021-3406-0 34552210

# Response

**Published:** 2022-08-05

**Authors:** Jagadeesha Pai B

**Affiliations:** Manipal Institute of Techhnology, India

**Response to Reviewers**

 05.08.2022

**Respected Reviewers,**

 We would like to thank you for the opportunity to make revisions in our manuscript entitled “Mapping accessibility to oral health care in coastal India – A geospatial approach using a geographic information system”. We would also like to thank the reviewer for his comments and suggestions on our manuscript.

 We have responded to each comment by the reviewer below:

**Comment: **In the introduction section, the authors introduce healthcare accessibility well but do not discuss the state of knowledge on dental care accessibility. Discuss previous works summarizing what similar studies (global, regional, local) have found. I have added references that may be helpful to the authors. 

**Response to reviewers: **We have now added that, sir

**Changes done on page number: **Page No. 4

**Comment: **I would recommend not using 1 sentence paragraphs in the manuscript. For example, "socioeconomic data" has only one sentence. You can either add a few more sentences to such subsections or combine them into a single section under appropriate headings. 

**Response to reviewers: **We have now done that sir

**Changes done on page number: **Page number 5

**Comment: **I would strongly encourage the authors to include a site map showing the location of the three districts with all the major cities clearly shown, and then also show a Karnataka map (with just district boundaries) within the Indian map (state boundaries only). Include the Arabian sea. 

**Response to reviewers: **Location map is included sir

**Changes done on page number: **Page number 5

**Comment: **Revise **Fig. 1** with the following changes: 

 - Fix the legend heading, i.e., " *Selected_points*" is unnecessary. Figures should be clean without underscores.

 - Under three districts on the legend, it is not clear what the blue polygon represents as it stands without a label.  

 - I would also include a very light base map of neighboring districts and the Arabian Sea for reference. 

**Response to reviewers: **Modification has been incorporated sir and map is modified, sir

**Changes done on page number: **Page number 9

**Comment: **In the methods, briefly describe what technique (E.g., Kernel) / tool (E.g., Point Density tool) was used for density/heat mapping analysis. 

**Response to reviewers: **The method we used is Kernel Density Estimation and same has been explained.

**Changes done on page number: **Page No. 9

**Comment: **It is not clear what software was used to run Pearson and Chi-square tests. Please include the appropriate citations. 

**Response to reviewers: **We have now mentioned sir

**Changes done on page number: **Page No. 6

**Comment: **I am not sure this paragraph fits into the scope of discussion. It would better fit in the methods section or perhaps even the introduction section where the study area is described.

 " Hence, in our study we tried to measure geographic accessibility by locating the availability of dental practitioners in the coastal districts of Karnataka using GIS. A geographic information system (GIS) is a computerized system, that is created to analyse and display geographically referenced information as a layered map 6. According to Padminee K et al., Karnataka has the largest number of Dental practitioners for any state in India (34,768).14 For our work, geo-coordinates of the public and private oral health centres were collected and this data was used for GIS analysis using the open-source software, QGIS 3.14."

**Response to reviewers: **Sir, we usually start with a small introductory paragraph in Discussion. Justifying the reason for conducting the study.

**Comment: **In your limitations, also acknowledge the differences that may exist in dental service preferences among urban and rural populations. This applies to both dentist and patient behaviors. 

**Response to reviewers: **We have now added sir.

**Changes done on page number: **Page No. 10

**Comment: **In your conclusion section, refer to the sentence I have provided below. The statement is too strong/broad. Perhaps, rephrase to state how these problems can be better addressed with appropriate government support, along with support from private and non-governmental stakeholders.

 "All the above mentioned problems can be solved with appropriate government support in key areas."

**Response to reviewers: **We have now rectified sir

**Changes done on page number: **Page No. 11

 We hope to have carried out revisions to the satisfaction of the reviewer. Please let us know if more revisions are required.

 Regards

# Peer review: Reviewer response for version 1

**DOI:** 10.5256/f1000research.79617.r135730

**Published:** 2022-05-16

**Authors:** Patrick Calvano Küchler

**Affiliations:** 1 Department of Geography, Rio de Janeiro State University (FEBF), Rio de Janeiro, Brazil

In the present study, an attempt was made to measure geographic accessibility to oral health in the Indian state of Karnataka. The proposal was to use geoprocessing methods and tools available in open source geographic information systems to analyze the spatial distribution of oral health facilities in public and private spheres in urban and rural regions. Geographically, the data used were latitude and longitude extracted from the addresses, which made it possible to geocode the data using OpenStreetMap as a basis. Descriptive statistical analyzes were performed with the creation of one main index: the dentist-population ratio D:P, in addition to its correlations with per capita income and number of clinics where Pearson's correlation coefficient was used.

 In addition to the location map of the oral health units, a density index map was produced. I believe that the authors could better explore the geoprocessing tools in future work, producing layer overlays or even thematic maps with the D:P index for each district, which would make it possible to carry out a deeper discussion of the results, making the research more relevant. I believe that the contribution of this article to the scientific society is important, with an impact on public health management, where it became evident that the understanding of spatial distributions is essential for a broad understanding of society, producing information that support decisions in the public health management.

Is the work clearly and accurately presented and does it cite the current literature?

Yes

If applicable, is the statistical analysis and its interpretation appropriate?

Yes

Are all the source data underlying the results available to ensure full reproducibility?

Yes

Is the study design appropriate and is the work technically sound?

Yes

Are the conclusions drawn adequately supported by the results?

Yes

Are sufficient details of methods and analysis provided to allow replication by others?

Partly

Reviewer Expertise:

Geographic Information Systems; Health Geography

I confirm that I have read this submission and believe that I have an appropriate level of expertise to confirm that it is of an acceptable scientific standard.
